# IL-6/GATA2/SERPINE1 pathway is implicated in regulating cellular senescence after acute kidney injury

**DOI:** 10.3389/fmolb.2025.1538526

**Published:** 2025-02-11

**Authors:** Hongshuang Su, Xiaoxi Lin, Ayinuer Paredong, Congcong Yao, Yan Zhang, Mengke Geng, Yuqian Guan, Lichao Gong, Feng Jiang, Qi Lv, Songtao Shou, Heng Jin

**Affiliations:** 1 Department of Emergency Medicine, Tianjin Medical University General Hospital, Tianjin, China; 2 Department of Emergency and Critical Care Medicine, Renji Hospital, Shanghai Jiao Tong University School of Medicine, Shanghai, China; 3 Department of Critical Care Medicine, Hotan District People’s Hospital, Xinjiang, China; 4 Department of Ophthalmology, Tianjin Medical University General Hospital, Tianjin, China; 5 Institute of Disaster and Emergency Medicine, Tianjin University, Tianjin, China

**Keywords:** rhabdomyolysis-induced acute kidney injury, IL-6, SERPINE1, GATA2, cellular senescence

## Abstract

**Purpose:**

Acute kidney injury (AKI) secondary to Rhabdomyolysis syndrome represents a life-threatening complication, characterized by notably high incidence and mortality rates. The role of cellular senescence in the progression of AKI has increasingly garnered attention in recent years. Our previous research has demonstrated that remote ischemic postconditioning (RIPC) can attenuate renal cellular senescence and elevation of serum level of interleukin-6 (IL-6) induced by ischemia-reperfusion injury following crush injury. The objective of this study is to investigate the specific role of IL-6 in Rhabdomyolysis-induced AKI (RM-AKI).

**Methods:**

We established a mouse model of RM-AKI by intramuscular injection of glycerol and simulated RM-AKI at the cellular level by treating Hk-2 cells with myoglobin. Tocilizumab (TCZ), a humanized monoclonal antibody against the interleukin-6 (IL-6) receptor, is a key substance. IL-6, a multifunctional cytokine, plays a crucial role in the occurrence and development of various kidney diseases. It can promote inflammatory responses, cell proliferation, fibrosis, and other processes. TCZ exerts a protective effect on the kidneys by specifically binding to the IL-6 receptor and blocking the signal transduction of IL-6. Additionally, the levels of IL-6 were detected by employing ELISA kits. RNA sequencing analysis was performed on cells treated with myoglobin and tocilizumab. Flow cytometry was utilized to assess cell cycle distribution and the percentage of senescent cells. The expression levels of SERPINE1, GATA2, p53, and p21 were determined by real-time quantitative PCR and Western blot. Additionally, a dual-luciferase reporter gene assay was conducted to validate the binding effect of SERPINE1 and GATA2.

**Results:**

Transcriptome Analysis revealed that genes including GATA2 and SERPINE1 were downregulated in HK-2 cells following tocilizumab treatment. Inhibition of the IL-6 receptor by tocilizumab in these cells led to a reduction in cellular senescence, accompanied by decreased of the cell cycle regulatory proteins P53 and P21 in mRNA and protein levels, while alleviating cell cycle arrest. Additionally, a dual-luciferase reporter assay confirmed that GATA2 binds to the promoter of SERPINE1 (PAI-1), thereby initiating its transcription.

**Conclusion:**

The IL-6/GATA2/SERPINE1 pathway mediates cellular senescence after acute kidney injury, and inhibiting IL-6 can alleviate AKI-induced cellular senescence, providing an important basis for exploring new therapeutic strategies.

## 1 Introduction

Rhabdomyolysis syndrome (RM) is a condition characterized by shock and renal injury following skeletal muscle trauma, which is described as reperfusion injury resulting from traumatic rhabdomyolysis (Gonzalez, 2005; Sever et al., 2020). The kidney is often one of the primary organs affected in RM, and acute kidney injury (AKI) represents one of its most severe and life-threatening complications, with high incidence and mortality rates (Peiris, 2017; Zimmerman and Shen, 2014). Myoglobin is considered a major pathogenic factor in RM-associated AKI (RM-AKI) (Sever et al., 2011; Wang et al., 2021). The current management strategies for acute kidney injury (AKI) primarily include adopting symptomatic treatment measures to alleviate clinical symptoms, actively identifying and eliminating the underlying causes, while providing fundamental treatment for the primary disease, and timely employing dialysis therapy to support or replace renal function when kidney function is severely impaired. Previous experiments have demonstrated that ischemic postconditioning can reduce serum myoglobin and IL-6 levels in rabbits with crush injury, while also attenuating renal cellular senescence and tubular damage (Remote ischemic postconditioning protects against, 2022).

Cellular senescence is an irreversible process of cell cycle arrest, closely associated with the development of AKI. It plays a crucial role, particularly in the progression from AKI to chronic kidney disease (CKD) (Kumari and Jat, 2021). Chronic senescence is intimately linked to the senescence-associated secretory phenotype (SASP) and senescence-associated pathways. Based on our earlier research, we hypothesize that senescence contributes to the adverse outcomes of RM, including renal injury progressing to chronic kidney disease, renal failure, and persistent renal dysfunction (Lazzeri, 2021; Bae et al., 2020; Canaud et al., 2019).

The SASP plays a pivotal role in enhancing the effects of senescence (Jin et al., 2019). IL-6, as a representative of the SASP, is involved in immune regulation and oncogenesis, and may also inhibit tumorigenesis through cellular senescence (Sapochnik et al., 2017). The effects of IL-6 are primarily mediated through the IL-6/signal transducer and activator of transcription 3 (STAT3) pathway (Kojima et al., 2013; Johnson et al., 2018). Given that our previous experiments revealed that remote ischemic postconditioning improved renal injury, slowed senescence, and decreased serum IL-6 levels in rabbits with RM-AKI, this study aims to investigate the mechanisms underlying the role of IL-6 in RM-AKI-induced renal cellular senescence by simulating RM-associated renal injury *in vitro* and administering an IL-6 receptor blocker (Zager and Burkhart, 1997).

## 2 Materials and methods

### 2.1 Establishment of a mouse model of acute kidney injury induced by rhabdomyolysis

Using male C57BL/6 mice aged 6–8 weeks with a body weight of 18–22 g, the animals were acclimated for 1 week prior to the experiment, with the ambient temperature maintained at a suitable range (22°C ± 2°C) and relative humidity at a moderate level (50%–60%) (Liu et al., 2022; Chen and Hou, 2024). The experimental mice were randomly divided into two groups: a control group and an injury group. In the injury group, each mouse’s hind limbs were injected with a 50% glycerol solution (Glycerol, GL; SIGMA, G5516, USA) at a dose of 6 mL/kg body weight, administered via an equal-volume injection (Liu et al., 2022; Huang et al., 2017). Correspondingly, the hind limbs of mice in the control group received an equal volume of normal saline (Normal Saline, NS; OTSUKA, 2E77F3, China) injection, also at a dose of 6 mL/kg body weight. To ensure the reliability and statistical significance of the experimental results, each experimental group comprised at least three mice as samples (Zhang et al., 2015). Renal tissues were collected from the mice for subsequent experiments. The feeding and use of experimental animals both comply with the relevant regulations of the Animal Protection and Use Committee of Tianjin Medical University. The mice were euthanized by cervical dislocation in accordance with the relevant regulations and ethical guidelines of the Animal Protection and Use Committee of Tianjin Medical University.

### 2.2 Periodic Acid-Schiff (PAS) staining and Masson’s trichrome staining

Histological analysis was conducted on 4-μm (μm) thick paraffin-embedded sections of renal tissue using Periodic Acid-Schiff (PAS) staining and Masson’s Trichrome Stain (Jin et al., 2022). Adhering strictly to the principles of double-blinding and without knowledge of the sample origins. All specimens underwent comprehensive pathological evaluation, focusing on the infiltration of inflammatory cells, the degree of edema and necrosis in renal tubular epithelial cells, the fibrotic condition of the kidney tissue, and the presence of hyaline casts. On PAS-stained sections, observation fields were randomly selected in the renal cortex using a ×40 microscope. The scoring of tubular injury was based on the percentage of injured tubules relative to the total number of tubules: a score of 0 was assigned for 0% injury, representing normal tissue without damage; a score of one for less than 20% injury; a score of two for 20%–40% injury; a score of three for 40%–60% injury; and a score of four for more than 60% injury, indicating severe damage. Additionally, quantitative analysis was conducted on each Masson-stained section to accurately calculate the proportion of positive fibrotic areas, further assessing the degree of renal fibrosis.

### 2.3 Measurement of serum creatinine (Scr) and blood urea nitrogen (BUN) concentrations

The concentrations of Scr and BUN were quantitatively determined utilizing a state-of-the-art automated biochemical analyzer (model Chemray 800, manufactured by Rayto, China).

### 2.4 Enzyme-linked immunosorbent assay (ELISA)

IL-6 levels in the cell supernatants were determined using an Enzyme-Linked Immunosorbent Assay (ELISA) kit (elabscience, Wuhan, China), following the manufacturer’s instructions. For all samples, a standard curve was constructed according to the standards provided by the manufacturer.

### 2.5 Cell culture

All cell experiments were conducted using HK-2 cells, an immortalized proximal tubule cell line derived from normal adult humans [26]. The HK-2 cells were purchased from EK-Bioscience Biotechnology Co. (CC Y1234). The cells were cultured *in vitro* in Dulbecco’s Modified Eagle Medium/F12 (DMEM/F12, Invitrogen, C11330500BT) supplemented with 10% fetal bovine serum (FBS, Cellmax SA211.02) and penicillin-streptomycin (PS, Invitrogen, 15140122) and maintained under adherent culture conditions at 37°C with 5% CO2. When the cell density reached 80%, the cells were detached and passaged using 0.25% trypsin (Trypsin-EDTA, Invitrogen, 25200072). After 3-4 passages, the cells were seeded for experimentation.

HK-2 cells were treated with ferrous myoglobin *in vitro* to simulate a RM-AKI model, as only the reduced form of myoglobin (Fe2+) has been found to be cytotoxic. A stock solution of 40 mg/mL horse skeletal muscle myoglobin (Sigma M0630) was prepared in DMEM/F12. To this stock solution, 12 mM ascorbic acid (Sigma, A5960) was added, and the metmyoglobin (Fe3+) was reduced to myoglobin (Fe2+) by incubating at room temperature for 1 hour. The final concentration of myoglobin (Fe2+) was 150 μM, while the concentration of ascorbic acid was 2 mM [26]. Myoglobin (Fe2+) was then added to the HK-2 cells, which were subsequently incubated at 37°C with 5% CO_2_ for 24 h.

After the cells were exposed to myoglobin (Fe2+) for 24 h, the culture medium was aspirated, and the cells were washed three times with phosphate-buffered saline (PBS). Subsequently, 100 μg per milliliter of tocilizumab was added.

### 2.6 Cell cycle analysis

Cell cycle analysis was performed using a Cell Cycle Analysis Kit (Beyotime C1052) according to the manufacturer’s instructions. The HK-2 cells were digested and fixed overnight with pre-cooled 70% ethanol at 4°C, then resuspended in ice-cold PBS. The cells were stained with a staining solution containing RNase A and propidium iodide (PI) at 37°C for 30 min. Red fluorescence was detected by flow cytometry at an excitation wavelength of 488 nm. The DNA content of the cells and the number of cells in different phases of the cell cycle were analyzed using FlowJo software.

### 2.7 Quantitative detection of senescent cells

The detection is based on the hydrolysis of a membrane-permeable molecule, 5-dodecanoylaminofluorescein di-β-D-galactopyranoside (C12FDGlpBio, GC43021), which is taken up by cells and hydrolyzed by β-galactosidase in the lysosomes of senescent cells, emitting green fluorescence upon laser excitation for quantitative detection of senescent cells. Briefly, cells were treated with bafilomycin A1 (GlpBio, GC17597) at a final concentration of 100 nM to neutralize the acidic pH of lysosomes. The cells were then incubated at 37°C with 5% CO_2_ for 1 h. C12FDG was dissolved in pre-warmed fresh medium to a final concentration of 2 mM. Subsequently, C12FDG was added to the cells at a final concentration of 33 μM and incubated at 37°C with 5% CO_2_ for 2 h. Adherent cells were harvested using a trypsin solution and centrifuged at 1,000 *g* for 5 min at 4°C. The cell pellet was resuspended in 500 μL of cold PBS. The treated cells were then analyzed by flow cytometry, with thresholds set to distinguish between senescent and non-senescent cells [27].

### 2.8 Differential gene expression analysis and KEGG pathway enrichment analysis

RNA high-throughput sequencing was performed on cells treated with ferrous myoglobin and cells intervened with IL-6 receptor inhibitor to detect differentially expressed genes. Further analysis using the Kyoto Encyclopedia of Genes and Genomes (KEGG) was conducted to gain insights into the signaling pathways and biological functions involved in the differentially expressed genes between the RM-AKI group and the IL-6 receptor inhibitor group.

### 2.9 RNA extraction and quantitative real-time PCR (RT-qPCR) analysis

Total RNA was extracted using the RNA Purification Kit for Cells (TIANGEN, DP430), and cDNA was generated using the PrimeScript RT Kit (Takara) according to the manufacturer’s instructions. RT-qPCR experiments were conducted using SYBR Green qPCR Master Mix (Bimake, B21203) and the ABI7900HT Fast Real-Time PCR System (Applied Biosystems, CA, USA). GAPDH served as an internal control. Data were analyzed using the 2^-ΔΔCT method. Primer sequences are provided in Table 1.

**TABLE 1:** Primer sequences of related genes for reverse transcription quantitative polymerase chain reaction.

Genes	Primer sequence
P53	F: 5′‐TTCCTGAAAACAACGTTCTGTC‐3′ R: 5′‐AACCATTGTTCAATATCGTCCG‐3′
p21	F: 5′‐GATGGAACTTCGACTTTGTCAC‐3′ R: 5′‐GTCCACATGGTCTTCCTCTG‐3′
SERPINE1	F: 5′‐GGAGCGAGATCCCTCCAAAAT‐3′ R: 5′‐CAATCTTGAATCCCATAGCTGC‐3′
GAPDH	F: 5′‐GGAGCGAGATCCCTCCAAAAT‐3′ R: 5′‐GGCTGTTGTCATACTTCTCATGG‐3′
GATA2	F: 5′‐AAGGCTCGTTCCTGTTCAGAAG‐3′ R: 5′‐CCCATTCATCTTGTGGTAGAGG‐3′

### 2.10 Western blot assay

HK-2 cells were collected and lysed in Radio Immunoprecipitation Assay (RIPA) buffer (Meilunbio, Dalian, China) supplemented with Phenylmethanesulfonyl fluoride (PMSF) protease inhibitor cocktail (Meilunbio, Dalian, China). Equal amounts of protein lysates were separated on SDS-PAGE gels and subsequently transferred to PVDF membranes (Millipore). The membranes were blocked with 5% non-fat milk at room temperature for 1 h, followed by overnight incubation with primary antibodies at 4°C. Next, the membranes were incubated with secondary antibodies at room temperature for 1 h. The immunoreactive signals were visualized using an Enhanced Chemiluminescence Kit (Amersham Biosciences, Uppsala, Sweden).

The antibodies used for the Western blot experiments were as follows: anti-PAI-1 (Proteintech 13801-1-AP), anti-GATA2 (Proteintech, 11103-1-AP), anti-P53 (Proteintech, 10442-1-AP), anti-P21 (Proteintech, 10355-1-AP), anti-β-actin (Proteintech, 66009-1-Ig), goat anti-rabbit IgG-HRP (Absin, abs20040), and goat anti-mouse IgG-HRP (Absin, abs20039).

### 2.11 Dual-luciferase reporter assay

Luciferase activity assays were performed using the Dual-Luciferase Reporter Assay Kit (Promega, E1910) according to the manufacturer’s instructions. The 3′UTR fragments of both wild-type and mutant SERPINE1 genes were cloned into the pGL3-basic luciferase reporter vector (Promega). HEK293T cells (Sangon Biotech) were seeded into 24-well plates and co-transfected with the reporter plasmids and GATA2 mimiRM (Sangon Biotech) using Lipofectamine™ 2000 (Invitrogen) after 6 h of culturing. Luciferase activity was measured 48 h post-transfection using the Dual-Luciferase Reporter Assay Kit (Promega).

### 2.12 Senescence associated β-galactosidase (SA-β-gal) staining

Renal tissue sections embedded in OCT compound and fixed with paraformaldehyde were subjected to SA-β-gal staining. The tissue sections were stained overnight at 37°C using an SA-β-gal Staining Kit (Beyotime, China). Subsequently, the tissue sections were stained with Nuclear Fast Red (Servicebio, China) for 5 min at room temperature.

### 2.13 Statistical analysis

All data were analyzed using GraphPad Prism nine software. A paired t-test be used for cell culture studies with two groups, while one-way ANOVA was used for comparisons among multiple groups. In both cases, differences with P < 0.05 were considered statistically significant.

## 3 Results

### 3.1 The occurrence of renal injury and cellular senescence was observed *in vivo*

**FIGURE 1:**
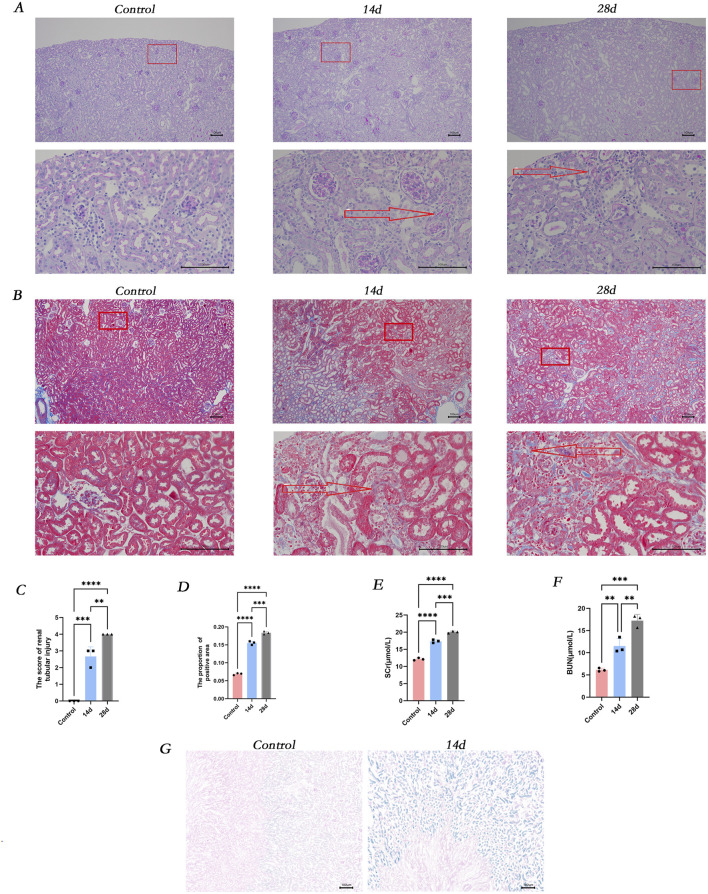
The occurrence of renal injury, fibrosis and cellular senescence was observed *in vivo*. **(A)** Masson-stained sections of mice kidneys. **(B)** PAS-stained sections of mice kidneys. **(C)** ImageJ was employed for digital image analysis of PAS-stained sections for quantitative assessment. **(D)** ImageJ was used for digital image analysis of Masson-stained sections for quantitative assessment. **(E)** The serum creatinine concentration in different groups of mice. **(F)** The blood urea nitrogen concentration in different groups of mice. **(G)** β-galactosidase staining of kidney tissues in various groups. ns, not significant. *P < 0.05, **P < 0.01, ***P < 0.001. Control: blank control group; 14d and 28d: mice at 14D and 28D after glycerol injection.

In the mice model of Crush syndrome-induced acute kidney injury (RM-AKI), at the observation time points of 14 and 28 days, the renal tissue exhibited a series of marked pathomorphological alterations. Specifically, there was evident deformation and necrosis of renal tubular epithelial cells, with the tubular structure appearing irregular and fragmented. Additionally, the expression of collagen in the renal interstitial area was significantly increased compared to the control group (Figures 1A, B). Compared with those in the control group, the renal function indices (Scr and BUN) were significantly greater in the RM-AKI group (Figures 1E–F). The SA-β-gal staining revealed that the expression of SA-β-gal was significantly elevated in the kidneys of RM-AKI mice (Figure 1G).

### 3.2 Myoglobin induces IL-6 secretion and cellular senescence *in vitro*

**FIGURE 2:**
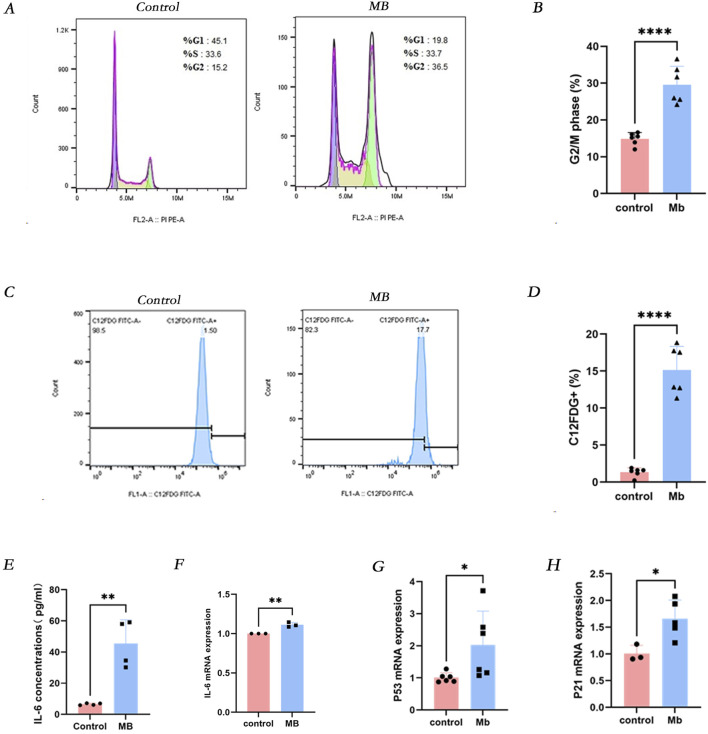
Myoglobin induces IL-6 secretion and cellular senescence *in vitro*. **(A)** The proportion of cells in the G2/M phase was observed to increase in the MB group by Flow cytometry, and the quantitative analysis of these proportions is presented in **(B)**. **(C)** The proportion of C12FDG + ratio of cells was observed to increase in the MB group through Flow cytometry, and the quantitative analysis of these proportions is presented in **(D)**. **(E)** The levels of IL-6 in cell supernatant were determined by using ELISA. **(F–H)** Relative mRNA expression of IL-6, p53 and p21 *in vitro* by qPCR. *P < 0.05, **P < 0.01, and ***P < 0.001. Control: blank control group. MB: Myoglobin treated group.

Based on previous experiments, we observed cellular senescence in renal tubular epithelial cells following crush injury in mice. To further validate this phenomenon, we established an *in vitro* model mimicking renal injury associated with crush syndrome. Flow cytometry analysis revealed a significant increase in the expression of senescence-associated β-galactosidase (SA-β-gal) in HK-2 cells treated with 150 micromolar (µM) myoglobin for 24 h (Figure 2C). Furthermore, we investigated whether cell senescence was correlated with cell cycle dysregulation and found that the proportion of cells in the G2/M phase was higher in all myoglobin-treated groups compared to the control group (Figure 2D). Renal tubular injury induced epithelial cell cycle arrest and senescence, accompanied by enhanced secretion of pro-inflammatory cytokines, particularly interleukin-6 (IL-6). Further examination of IL-6 levels showed a significant increase in IL-6 expression in the cell supernatant after myoglobin treatment (Figure 2E). These results suggest that myoglobin triggers cell senescence and G2/M phase arrest in HK-2 cells (Plovins et al., 1994). Additionally, qPCR and Western blot analyses were performed to detect the expression levels of the cell senescence markers p53 and p21. Compared to the control group, the mRNA and protein expression of p53 and p21 were significantly increased in cells treated with ferrous myoglobin (Figures 2G, H).

### 3.3 IL-6 receptor inhibitor (TCZ) alleviates cell senescence *in vitro*

**FIGURE 3:**
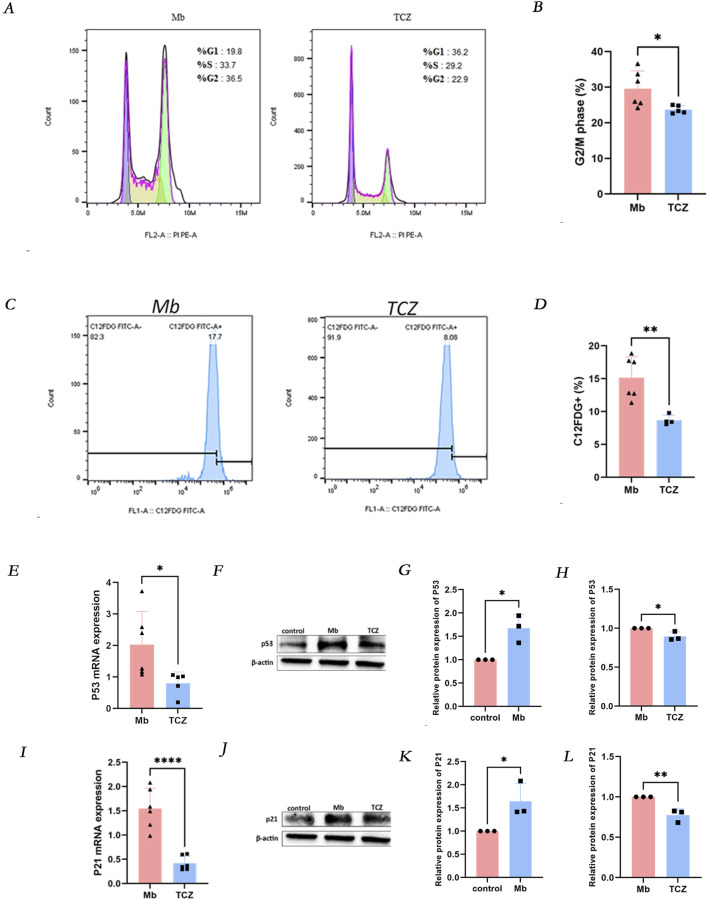
IL-6 receptor inhibitor (TCZ) reduces cellular senescence *in vitro*. **(A)** The proportion of cells in the G2/M phase was observed to decrease in the TCZ group by Flow cytometry, and the quantitative analysis of these proportions is presented in **(B)**. **(C)** The proportion of C12FDG + ratio of cells was observed to decrease in the TCZ group through Flow cytometry, and the quantitative analysis of these proportions is presented in **(D)**. **(E, I)** Relative mRNA expression of p53 and p21 *invitro* cells by qPCR. **(F)** The protein expression level of p53, and the relative band intensities were shown in **(G, H)**. **(J)** The protein expression levels of p21 and relative band intensity are shown in **(K, L)**. MB: Myoglobin treated group. TCZ: tocilizumab treated group.

After a 24-h intervention with the IL-6 receptor inhibitor tocilizumab, compared to the ferrous myoglobin group, the proportion of cells in the G2/M phase of the cell cycle was reduced in the tocilizumab-treated group (Figures 3A, B), and the percentage of SA-β-gal positive cells decreased (Figures 3C, D). Quantitative PCR (qPCR) and Western blot analyses were conducted to assess the expression levels of the cell senescence markers p53 and p21. The mRNA and protein levels of p53 and p21 were significantly lower in cells treated with the IL-6 receptor inhibitor compared to those in the ferrous myoglobin-treated group (Figures 3E–L). These findings indicate that cellular senescence was attenuated in cells treated with the IL-6 inhibitor compared to those exposed to ferrous myoglobin.

### 3.4 IL-6 receptor inhibitor attenuates cellular senescence via the SERPINE1 signaling pathway

**FIGURE 4:**
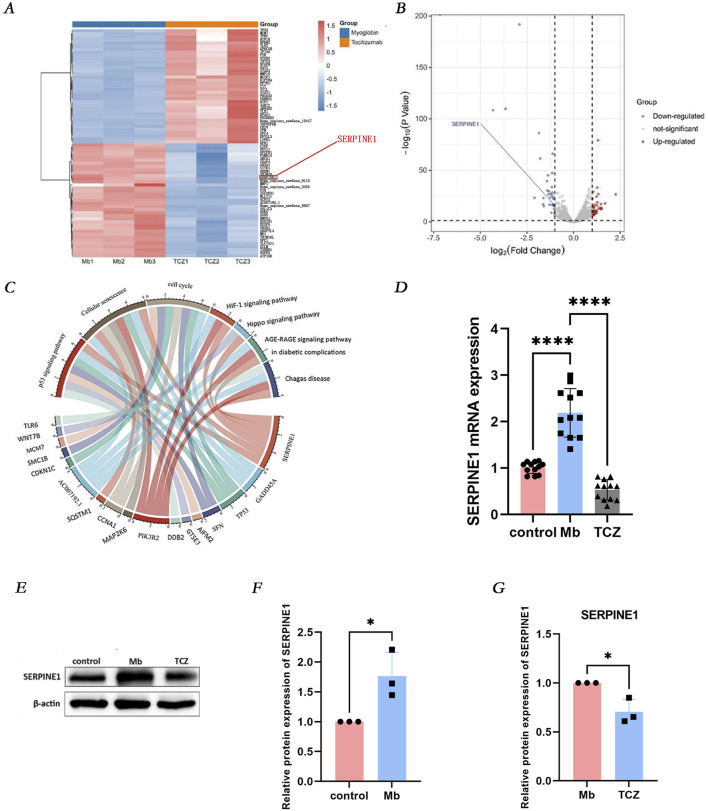
IL-6 receptor inhibitor attenuates cellular senescence via the SERPINE1 signaling pathway. **(A, B)** RNA sequencing predicts differentially expressed genes (DEGs) between the Mb group and the TCZ group. **(C)** Identify the main enriched pathways of DEGs through Kyoto Encyclopedia of Genes and Genomes analysis. **(D)** Relative mRNA expression of SERPINE1 *in vitro* by qPCR. **(E)** The protein expression level of SERPINE1, and the relative band intensities were shown in **(F, G)**.

RNA high-throughput sequencing was performed on cells treated with ferrous myoglobin and those subjected to IL-6 receptor inhibitor intervention, resulting in the detection of 13,327 genes. Among these, 494 genes exhibited significant differential expression, comprising 339 upregulated genes (depicted in red) and 155 downregulated genes (depicted in blue) (Figures 4A, B). To further elucidate the signaling pathways and biological functions associated with the differentially expressed genes in the RM-AKI group and the IL-6 receptor inhibitor group, Kyoto Encyclopedia of Genes and Genomes (KEGG) analysis was conducted. The downregulated differentially expressed genes involved in the cell senescence pathway included PIK3R2, SERPINE1, MAP2K6, GADD45A, TP53, CCNA1, SQSTM1, and AC007192.1, with SERPINE1 playing a role in regulating the p53 signaling pathway, cell senescence, and cell cycle (Figure 4C). Validation by quantitative PCR (qPCR) and Western blot analysis revealed that, compared to the control group, SERPINE1 mRNA and protein expression were increased in cells treated with ferrous myoglobin, while IL-6 receptor inhibitor intervention significantly reduced SERPINE1 mRNA and protein expression in cells (Figures 4D–G). KEGG analysis indicated that SERPINE1 participates in the p53 signaling pathway (Figure 4C). In the second part of the experiment, qPCR and Western blot analysis demonstrated that IL-6 receptor inhibitor intervention led to a significant decrease in the mRNA and protein expression of p53 and p21 in RM-AKI cells (Figures 3E–L). These findings suggest that in the *in vitro* RM-AKI model, SERPINE1 may mediate HK-2 cell senescence through regulation of the p53/p21 pathway.

### 3.5 GATA2 is upstream of SERPINE1 and regulates its expression

**FIGURE 5:**
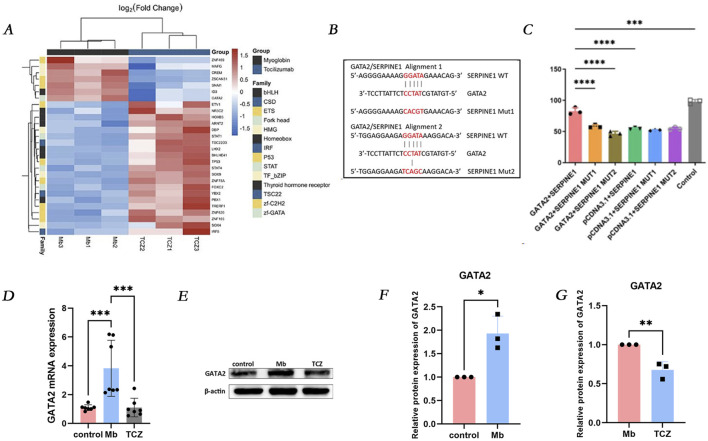
GATA2 is upstream of SERPINE1 and regulates its expression. **(A)** Heat map of all differentially expressed transcription factors between Mb and TCZ groups. **(B)** The binding site of GATA2 with SERPINE1. **(C)** Luciferase reporter assay was performed to examine the binding relationship between GATA2 and SERPINE1. **(D)** Relative mRNA expression of GATA2 in HK-2 cells by qPCR. **(E)** The protein expression level of GATA2, and the relative band intensities were shown in **(F, G)**.

In the RM-AKI group and the IL-6 receptor inhibitor group, 28 transcription factors exhibited significant differential expressions, including 21 upregulated genes (depicted in red) and seven downregulated genes (depicted in blue) (Figure 5A). Notably, GATA2 was significantly downregulated in the IL-6 receptor inhibitor group. Dual-luciferase reporter gene analysis demonstrated that in 293T cells, the transcription factor GATA2 exerted a positive regulatory effect on the SERPINE1 promoter. GATA2 was found to activate the SERPINE1, SERPINE1MUT1, and SERPINE1MUT2 promoters, albeit with a less pronounced effect on the latter two mutants. Following the mutation of SERPINE1MUT1 and SERPINE1MUT2, the positive regulatory effect of GATA2 on the SERPINE1 promoter was diminished, suggesting that GATA2 directly interacts with the SERPINE1 promoter to regulate SERPINE1 transcription (Figures 5B, C). Validation by quantitative PCR (qPCR) and Western blot analysis revealed that, compared to the control group, GATA2 mRNA and protein expression were significantly increased in cells treated with ferrous myoglobin, while IL-6 receptor inhibitor intervention led to a marked decrease in GATA2 mRNA and protein expression in cells (Figures 5D–G).

## 4 Discussion

This study demonstrates that by antagonizing the IL-6 receptor, the expression of GATA2, SERPINE1 (also known as plasminogen activator inhibitor-1, PAI-1), p53, and p21 is downregulated, thereby alleviating myoglobin-induced senescence in HK-2 cells and improving cell cycle arrest (Eren et al., 2014; Kang et al., 2020). These findings suggest that IL-6 may activate the p53/p21 pathway via GATA2/SERPINE1, inducing senescence in renal tubular epithelial cells during RM-AKI. SERPINE1/PAI-1 regulates involuntary proteolytic cascades in cells, which are crucial determinants of aging *in vivo* (Gifford et al., 2021). While PAI-1 is typically produced in trace amounts in healthy kidneys, its production is markedly increased in various acute and chronic kidney diseases (Sylwia et al., 2013). Elevated PAI-1 expression leads to extracellular matrix (ECM) accumulation in diabetic nephropathy, chronic kidney disease, hemodialysis, peritoneal dialysis, and kidney transplantation. In severe COVID-19 patients, IL-6 signaling regulates PAI-1 production, forming a positive feedback loop that exacerbates systemic inflammation and circulatory deterioration, which can be mitigated by blocking the IL-6 receptor. Previous studies have shown that sustained expression of PAI-1 (SERPINE1) results in an increased percentage of cells in the G2/M phase, accompanied by a decrease in the G1 phase, elevated levels of the G2/M arrest marker P-histone H3, upregulation of profibrotic cytokines, and ECM deposition (Gire and Tomas, 2021). These findings are consistent with our observations, where HK-2 cells treated with myoglobin exhibited an increased number of cells in the G2/M phase, whereas those treated with tocilizumab showed a reduction. Similarly, studies have implicated a PAI-1-Klotho-p53-dependent mechanism in tubular epithelial cell cycle arrest and fibrosis, with PAI-1 overexpression increasing total p53 protein levels. These theories further support our experimental findings (Gifford et al., 2021).

GATA2, a zinc finger-containing transcription factor, plays a pivotal role in various organ developments, including kidney development, and is highly expressed in primitive tissues during early embryonic development (Ainoya et al., 2012). Studies have shown that GATA2 mediates renal injury after ischemia-reperfusion by promoting the release of inflammatory cytokines from renal collecting duct CD cells. In GATA2-knockdown CD cells, the expression of inflammatory cytokine genes is attenuated, and IRI-induced renal injury is ameliorated. Overexpression of GATA2 in mouse 2-cell embryos leads to cell cycle arrest (Yu et al., 2017). We confirmed through dual-luciferase reporter gene experiments that the transcription factor GATA2 indeed acts on the SERPINE1 promoter. Combining these findings on GATA2 and SERPINE1, we hypothesize that IL-6 may activate the p53/p21 pathway via GATA2/SERPINE1, thereby inducing senescence in renal tubular epithelial cells during RM-AKI (Komatsu et al., 2021).

Although HK-2 cells are widely used as a surrogate for renal tubular epithelial cells, they may not fully recapitulate the complex physiological and pathological responses observed *in vivo*. Further validation in human samples is crucial. While cell line studies provide useful information, they cannot replicate the biological complexity of human tissues. Analyzing primary renal cells or tissue biopsies from patients with relevant diseases will provide a more physiologically relevant context for our findings. Such validation will help confirm whether the observed effects are applicable to human diseases and may guide the development of potential therapeutic strategies. Additionally, extending our research to other animal models, such as mice or rats, will offer additional insights into the *in vivo* relevance of our results. Our current study provides valuable preliminary data, and recognizing its limitations is key to addressing these issues. In the future, we aim to enhance the generalizability of our research by validating our findings in human samples and other animal models, thereby making more meaningful contributions to the understanding and treatment of renal diseases (Cahu and Sola, 2013). Notably, tocilizumab (TCZ), a biologic agent widely used in the treatment of rheumatic diseases, is well-recognized for its anti-inflammatory effects through specific blockade of the IL-6 receptor. Despite limited research in the renal field, our study provides a strong theoretical basis for TCZ as a potential therapeutic agent for AKI-induced cell senescence (Jin et al., 2020). The expression levels of IL-6, GATA2, and SERPINE1 can serve as biomarkers for AKI severity and prognosis, aiding in diagnosis and personalized treatment planning. IL-6 receptor antagonists like tocilizumab can reduce cellular senescence and improve renal function, but optimal dosing and patient responsiveness need clinical trials to determine. Long-term use safety and efficacy of IL-6 inhibitors also require further study.

**FIGURE 6:**
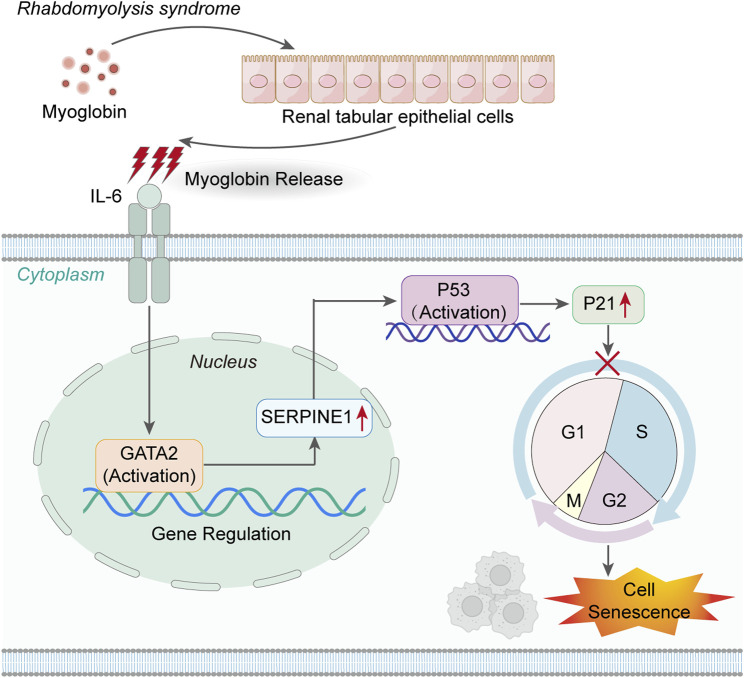
IL-6 activates the GATA2/SERPINE1/p53/p21 signaling axis, mediating the senescence of renal tubular epithelial cells.

In summary, this study not only unveils a novel mechanism of IL-6/GATA2/SERPINE1-mediated cell senescence in acute kidney injury but also provides preliminary evidence for TCZ as an effective treatment for AKI-induced cell senescence (Figure 6). In the future, we anticipate further validation of this hypothesis through additional *in vivo* and *in vitro* experimental data, ultimately offering improved therapeutic prospects for patients with AKI.

## 5 Conclusion

Inhibition of the IL-6/GATA2/SERPINE1 pathway alleviates cellular senescence in acute kidney injury (AKI), suggesting that targeted suppression of IL-6 may represent a novel therapeutic strategy to mitigate AKI-induced cellular senescence, offering potential therapeutic prospects for patients with AKI.

## Data Availability

The original contributions presented in the study are included in the article/Supplementary Material, further inquiries can be directed to the corresponding author.
