# Supplementary material for: IL-6/GATA2/SERPINE1 pathway is implicated in regulating cellular senescence after acute kidney injury
Source: Front Mol Biosci. 2025 Feb 11;12:1538526. doi: 10.3389/fmolb.2025.1538526 (PMC11850272; doi:10.3389/fmolb.2025.1538526)
Supplement: Supplementary file 2 [file Image1.pdf]

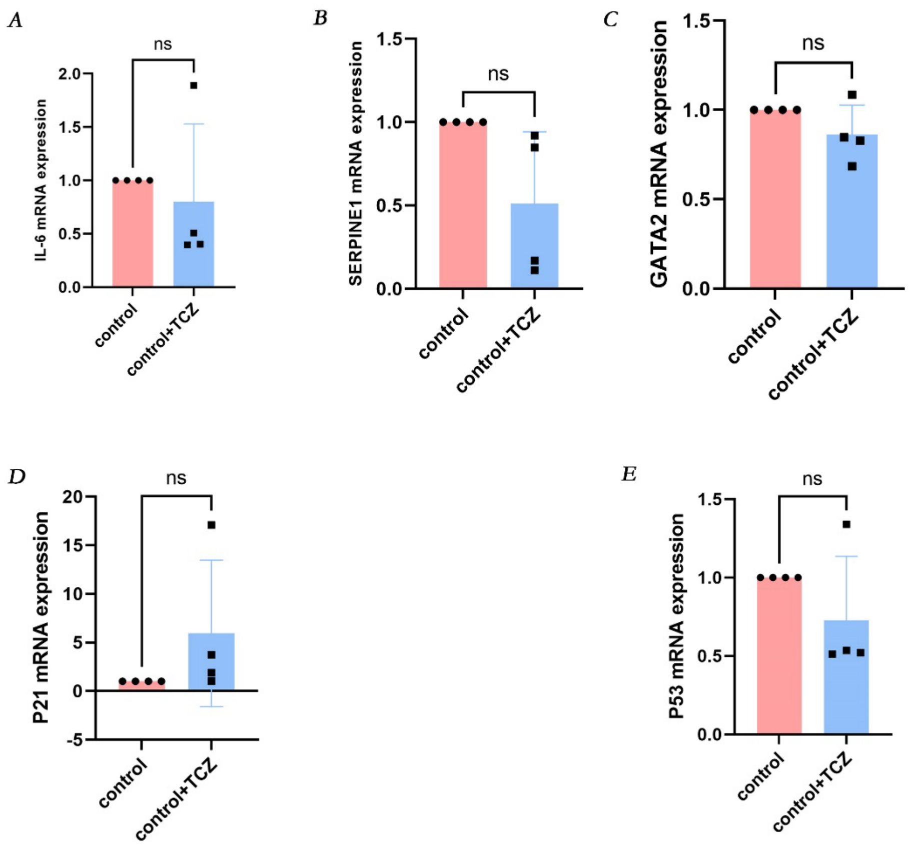

Figure S1 (A)Relative mRNA expression of IL-6 in vitro by qPCR. (B)Relative mRNA expression of SERPINE1 in vitro by qPCR. (C)Relative mRNA expression of GATA2 in vitro by qPCR. (D)Relative mRNA expression of p53 in vitro by qPCR. (E) Relative mRNA expression of p21 in vitro by qPCR. ns, not significant. * $P < 0.05$, ** $P < 0.01$, *** $P < 0.001$. control: blank control group; control+TCZ: treatment control group.
